# Mediating role of inflammatory markers in the relationship between cotinine levels and total bone mineral density

**DOI:** 10.1371/journal.pone.0329062

**Published:** 2025-08-08

**Authors:** Lei Huang, Xianghong Wang, Xianxu Zhang, Shicheng Li, Xin Liu, Zhong Ma, Bin Qian, Changlin Zhou, Zhiqiang Luo

**Affiliations:** 1 Department of Orthopaedics, Lanzhou University Second Hospital, Lanzhou, Gansu, China; 2 Department of Orthopaedics, Gansu Jiuquan Hospital of Traditional Chinese Medicine, Jiuquan, Gansu, China; University of Vermont, UNITED STATES OF AMERICA

## Abstract

**Objectives:**

This study investigates the relationship between smoking and total BMD and examines the mediating role of inflammatory markers in this relationship.

**Method:**

In total, 22,022 participants were included in this study, based on data from the National Health and Nutrition Examination Survey for the periods 2001–2006 and 2011–2018. Weighted linear regression models and restricted cubic splines(RCS) were leveraged to examine the linear or nonlinear relationship between serum cotinine levels and total BMD. Additionally, mediation analysis was leveraged to appraise the potential mediating effects of inflammatory markers, such as lymphocytes, monocytes, neutrophils, and platelets, in the relationship between cotinine and total BMD.

**Results:**

After fully adjusting for all covariates, an increase of one unit in cotinine corresponded to a 0.00022 g/cm^2^ decrease in total BMD (Beta = −0.00022, 95% CI: −0.0003 ~ −0.0000, P = 0.0069). The RCS analysis indicated an “n-shaped” relationship between cotinine and total BMD (P-nonlinear = 0.0069). According to the mediation analysis, monocytes and neutrophils acted as mediators in the relationship between cotinine and total BMD, with mediation effects accounting for 19.8% and 19.6%, respectively.

**Conclusions:**

Smoking serves as a risk factor for reduced BMD, and the impact on BMD is partially mediated by inflammatory markers such as monocytes and neutrophils. Platelets can moderate the effect of cotinine on total BMD to some extent.

## Introduction

Osteoporosis (OP) is a chronic, systemic skeletal disease with high prevalence, marked by a reduction in total bone mineral density (BMD), decreased bone mass [1], deterioration of bone tissue microstructure, and elevated bone fragility [2]. Based on epidemiological statistics, OP affects nearly 2 billion individuals globally [3]. In a 2018 meta-analysis, it was estimated that the global prevalence of OP is between 5% and 37% [4]. About 1/3 of women and 1/5 of men over 50 have an osteoporotic fracture during their lifetime [5]. Additionally, a study indicates that by 2025, the direct costs related to OP will soar to $25.3 billion per year [6], placing a tremendous economic burden on society. Thus, OP has emerged as an urgent global public health concern.

As outlined by the World Health Organization, BMD measured with dual-energy X-ray absorptiometry (DXA) is the gold standard in diagnosing OP and is essential for OP prevention and early detection [7]. Nevertheless, the factors contributing to the decrease in BMD are multifaceted, including controllable factors like smoking, drinking, and exercise, as well as uncontrollable factors like sex, age, race, and genetics [8,9]. Cotinine is a significant metabolic byproduct formed during the decomposition of nicotine in the process of smoking. Cotinine levels in the serum can indicate recent nicotine exposure from tobacco smoke, offering a more visual and objective way to assess the smoking status of patients [10,11]. By elevating superoxide free radicals and decreasing intracellular glutathione in mesenchymal stem/stromal cells, cotinine induces oxidative stress, which is harmful to osteogenic differentiation [12]. It also impacts bone resorption, resulting in an imbalance in bone remodeling and then leading to a decline in BMD [13]. Research in osteoimmunology reveals that inflammation is a key contributor to OP pathogenesis and fragile bone fractures [14]. Smokers exhibit higher levels of interleukin (IL)-1β (IL-1β), IL-6, and tumor necrosis factor α (TNF-α) in their bone tissue compared to non-smokers [15,16]. This indicates that inflammation could be a potential mediator in the pathway through which cotinine influences BMD. Thus, this study aims to explore the mediating effects of inflammatory markers (lymphocytes [LYMs], monocytes [MONs], neutrophils [NEUs], platelets [PLTs]) in the relationship between smoking and total BMD, following the investigation of their associations. To our knowledge, up until the start of this study, no published articles have investigated the mediating effect of inflammatory markers in the relationship between cotinine and total BMD.

## Material and methods

### Study population

This study leveraged the National Health and Nutrition Examination Survey (NHANES) database (http://www.cdc.gov/nchs/nhanes.htm) from the U.S. The study is a research project led by the National Center for Health Statistics (NCHS), with the goal of reflecting the health and nutrition status of the U.S. population [17]. This study was carried out in accordance with the Declaration of Helsinki and utilized NHANES data that underwent ethical review by NCHS. As a secondary analysis of an existing approved dataset conducted following the STROBE guidelines (S1 File), no additional ethical approval was required.

**Fig 1:**
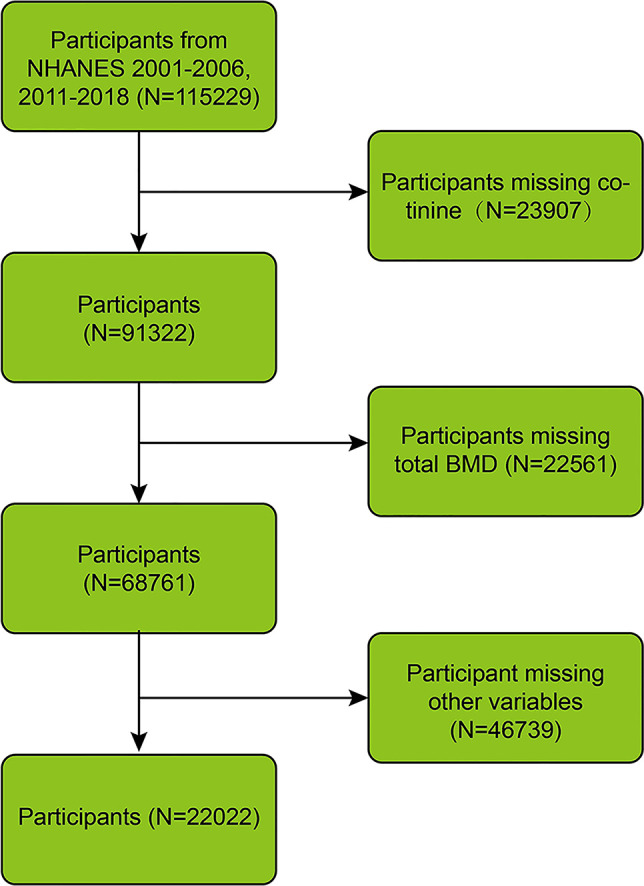
Flowchart of the participants’ selection.

Data from seven cycles of NHANES, covering the periods 2001–2006 and 2011–2018, were leveraged, involving 115,229 participants in total. Missing data were excluded, including for cotinine (n = 23,907), total BMD (n = 22,561), body mass index (BMI) (n = 935), education level (n = 45,507), and serum inflammatory markers (n = 297). Ultimately, 22,022 participants were included (Fig 1).

### Study variables

The primary outcome was total BMD, which was calculated based on the results from DXA. Cotinine was themain exposure variable. Cotinine levels in serum were measured by an isotope-dilution high-performance liquid chromatography-atmospheric pressure chemical ionization tandem mass spectrometry (ID HPLC-APCI MS/MS) method. Initially, methyl-D3-cotinine was incorporated into the serum samples to serve as an internal standard. Following alkalinization, the samples underwent pretreatment with a solid-phase liquid extraction plate. The next step involved extracting the samples with an isopropanol/dichloromethane mixture, followed by injection into a C18 HPLC column for separation. Finally, the extract was monitored via APCI-MS/MS, with the m/z 80 daughter ion of the m/z 177 quasi-molecular ion employed for identifying cotinine [18]. Additionally, the covariates incorporated were sex, race, age, BMI, and education level.

### Statistical analysis

Due to the intricate survey design of NHANES, new weights for the survey data were calculated according to the analysis guidelines edited by NCHS. For continuous variables, the mean and standard error were leveraged, whereas categorical variables were presented as percentages. Weighted linear regression was applied, utilizing two adjusted models to examine the relationship between cotinine and total BMD. No adjustments were made in Model 1, and Model 2 was adjusted for sex, age, race, BMI, and other covariates. A restricted cubic spline (RCS) analysis was leveraged to appraise the potential nonlinear association of cotinine with total BMD after adjusting for covariates. Nonlinearity was appraised through a likelihood ratio test. Mediation analysis was leveraged to examine the mediating role of inflammatory markers, such as LYMs, MONs, NEUs, and PLTs, in the relationship between cotinine and total BMD. All statistical analyses were carried out by means of R software (version 4.4.1). The mediation analysis was executed utilizing the mediation package.

## Results

### Participant baseline characteristics

In total, 22,022 participants were included, with an average age of 42 years. Males and females each comprised 50% of the participants. As per a previous study, the cotinine concentration was divided into three quartile ranges: < 0.05, 0.05–2.99, and ≥3.00 [19]. The mean value of total BMD was 1.14 (0.12). Marked differences were noticed across sex, age, race, BMI, education level, and various inflammatory markers (all p < 0.05). Additionally, LYMs, MONs, NEUs, and PLTs were higher in groups with higher cotinine concentrations compared to the lowest cotinine group (Table 1).

**Table 1:** Characteristics of participants.

Characteristic	N^1^	Overall	Minimal Exposure	Low Exposure	High Exposure	p-value^3^
		N = 146,486,317^2^	N = 70,029,020^2^	N = 32,272,878^2^	N = 44,184,418^2^	
**BMI (kg/m**^**2**^)	22,022	29 (7)	29 (6)	29 (7)	28 (6)	<0.001
**LYM**	22,022	2.18 (1.03)	2.11 (1.11)	2.12 (1.14)	2.34 (0.75)	<0.001
**MON**	22,022	0.56 (0.19)	0.54 (0.18)	0.55 (0.18)	0.60 (0.19)	<0.001
**NEU**	22,022	4.32 (1.66)	4.14 (1.50)	4.12 (1.57)	4.76 (1.88)	<0.001
**PLT**	22,022	259 (65)	256 (64)	264 (67)	260 (67)	<0.001
**AGE (YEARS)**	22,022	42 (14)	44 (14)	42 (15)	40 (13)	<0.001
**GENDER,%**	22,022					<0.001
Male		11,037 (50%)	4,389 (43%)	2,631 (51%)	4,017 (60%)	
Female		10,985 (50%)	5,914 (57%)	2,599 (49%)	2,472 (40%)	
**Race,%**	22,022					<0.001
Mexican American		4,026 (9.1%)	2,343 (11%)	908 (9.2%)	775 (6.1%)	
Other Hispanic		1,505 (5.8%)	858 (6.7%)	321 (6.0%)	326 (4.3%)	
Non-Hispanic White		9,596 (67%)	4,367 (68%)	2,044 (62%)	3,185 (70%)	
Non-Hispanic Black		4,482 (11%)	1,392 (7.0%)	1,406 (15%)	1,684 (13%)	
Other Race		2,413 (7.3%)	1,343 (7.7%)	551 (7.9%)	519 (6.2%)	
**EDU,%**	22,022					<0.001
Less 9		2,186 (5.1%)	1,095 (4.8%)	569 (6.3%)	522 (4.5%)	
GRADE 9–11		2,974 (10%)	919 (5.9%)	747 (10%)	1,308 (17%)	
HIGH SCHOOL		5,056 (24%)	1,805 (17%)	1,304 (27%)	1,947 (32%)	
COLLEGE		11,806 (61%)	6,484 (72%)	2,610 (57%)	2,712 (46%)	
**TOBMD(g/cm**^**2**^)	22,022	1.14 (0.12)	1.13 (0.11)	1.15 (0.12)	1.15 (0.12)	<0.001

^1^ N not Missing (unweighted)

^2^ Mean (SD); n (unweighted)(%)

^3^ Design-based KruskalWallis test; Pearson’sc2: Rao & Scott adjustment

### Linear regression

According to the multiple linear regression model, in Model 1, each one-unit elevation in cotinine led to a 0.00026 g/cm^2^ reduction in total BMD (Beta = −0.00026, 95% confidence interval [CI]: −0.0004 ~ −0.0001, P = 0.0012). In Model 2, an increase of one unit in cotinine was tied to a reduction of 0.00022 g/cm^2^ in total BMD (Beta = −0.00022, 95% CI: −0.0003 ~ −0.0000, P = 0.0069) (Table 2).

**Table 2:** Association between cotinine and total bone mineral density.

MODEL1 (Unadjusted)		
**EXPOSURE**	**β (95% CI)**	***P***
Cotinine	−0.000 (−0.0004, −0.0001)	0.0012
**MODEL2 (adjusted)**		
**EXPOSURE**	**β (95% CI)**	***P***
Cotinine	−0.000 (−0.0003, −0.0000)	0.0069
AGE	−0.001 (−0.0016, −0.0013)	<0.0001
**RACE**		
Mexican American	**Ref**	**Ref**
Other Hispanic	0.003 (−0.0052, 0.0120)	0.44
Non-Hispanic White	0.036 (0.0288, 0.0432)	<0.0001
Non-Hispanic Black	0.094 (0.0846, 0.1034)	<0.0001
Other Race	0.010 (0.0006, 0.0194)	0.030
BMI	0.002 (0.0023, 0.0030)	<0.0001
**GENDER**		
MALE	**Ref**	**Ref**
FEMALE	−0.084 (−0.0873, −0.0807)	<0.0001

Model 1: no covariates were adjusted.

Model 2: Adjusted for age, sex, race, BMI.

### RCS analysis

**Fig 2:**
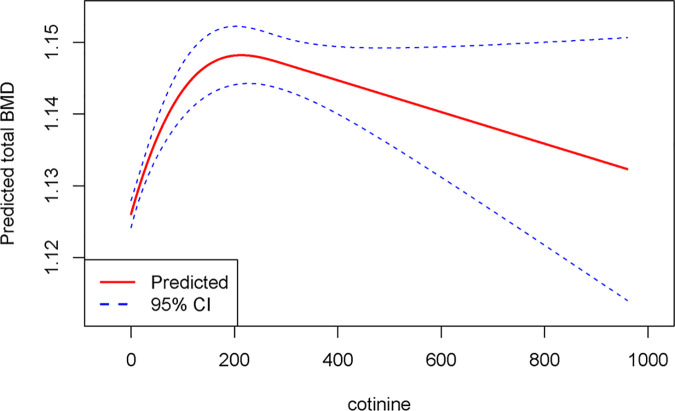
RCS analysis of the association between cotinine and total bone mineral density.

The RCS analysis indicated a noticeable nonlinear “n-shaped” association between cotinine and total BMD (P-nonlinear = 0.0069). The negative effect of cotinine on total BMD was not notable when the cotinine concentration in the body was below 192.98 ng/ml. However, as cotinine levels exceeded 192.98 ng/ml, the effect on total BMD became noticeable (Fig 2).

### Mediation analysis

**Fig 3:**
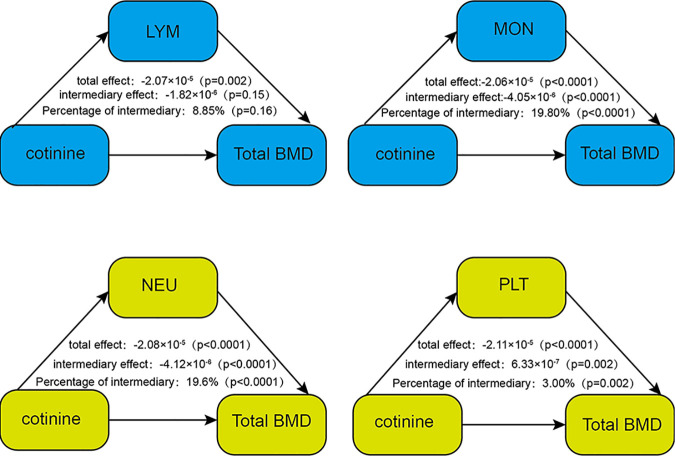
Mediating role of inflammatory markers in the association between cotinine and total bone mineral density.

According to the mediation analysis, LYMs had no mediating effect on the relationship between cotinine and total BMD (P > 0.05, mediation effect = −1.82 × 10^−6^). MONs and NEUs mediated the relationship between cotinine and total BMD (both P < 0.05), with mediation proportions of 19.8% (mediation effect = −4.05 × 10^−6^) and 19.6% (mediation effect = −4.12 × 10^−6^), respectively. Although PLTs also exhibited a mediating effect (P < 0.05), its direction was opposite (mediation effect = 6.33 × 10^−7^). PLTs can moderate the effect of cotinine on total BMD to some extent (Fig 3).

## Discussion

This is the first study to investigate the relationship between serum cotinine concentrations and total BMD, and to explore the potential mediating role of inflammatory markers. The results demonstrated a relationship between increased serum cotinine levels and decreased total BMD, characterized by a significant non-linear ‘n-shaped’ association (P non-linear < 0.001), with a critical inflection point at 192.98 ng/ml. Further analyses showed that blood cell inflammatory markers (MON, NEU, and PLT) can mediate the cotinine-induced reduction in total BMD. These findings offer novel insights into the skeletal effects of smoking and highlight the importance of strengthening BMD monitoring in smokers, especially those with high serum cotinine concentrations. In clinical practice, our findings suggest that monitoring and regulating the levels of inflammatory markers may serve as a novel strategy for OP prevention and management in this population.

Earlier studies have demonstrated a relationship between higher serum cotinine levels and reduced lumbar spine BMD. Thus, cutting down on smoking can contribute to better bone health to a certain extent [20]. Moreover, research indicates that increased serum cotinine concentrations are linked to a decrease in trabecular bone score, and this association is more pronounced in women [21]. However, this study reveals that the impacts of cotinine on bone health are not confined to a particular area, but also negatively affect total BMD. From a mechanistic perspective, cotinine may influence bone density through multiple biological pathways, including inflammatory response [22], oxidative stress [23], ferroptosis [24], disruptions in vitamin D metabolism [25], alterations in bone metabolism [26], mitochondrial dysfunction [27], and disruption of sex hormones [28]. Thus, it is clear that inflammation is closely tied to bone remodeling. In comparison to non-smokers, smokers have increased white blood cell counts, with the count rising in proportion to nicotine levels [29]. Moreover, the inflammation triggered by smoking leads to an elevation in MON levels, which play a crucial role in the immune system. Smoking-induced activation of MONs stimulates the release of numerous inflammatory cytokines, such as IL-6, TNF-α, and IL-1β [30]. IL-6 can upregulate the expression of receptor activator of nuclear factor-κB ligand (RANKL). When RANKL binds to its receptor RANK, it can facilitate the differentiation and activation of osteoclasts [31], thereby enhancing bone resorption. Additionally, IL-6 activates the toll-like receptor (TLR) 2, TLR4, and protein kinase B pathways [32], contributing to the development of chronic inflammatory OP and suppressing the expression of β-catenin and Setd7 [33]. β-catenin, a key component of the canonical Wnt signaling pathway, is crucial for the osteogenic differentiation of bone marrow mesenchymal stem cells [34]. Inhibition of this pathway may impair bone metabolism. TNF-α plays a critical role in maintaining the dynamic balance between bone formation and resorption, thereby influencing both bone metabolism and bone-related immune regulation. It has been shown that TNF-α binding to TNF receptor 1 (TNFR1) can promote inflammatory responses. Atsttrin can inhibit TNF-α–induced osteoclastogenesis via TNFR1 [35]. Furthermore, IL-1β and IL-18 are associated with bone loss in postmenopausal women. Compared with healthy controls, whole blood cells from postmenopausal OP patients exhibit elevated levels of IL-1β, IL-6, TNF-α, interferon-γ (IFN-γ), and granulocyte-macrophage colony-stimulating factor following in vitro stimulation. Additionally, IL-1β levels are negatively correlated with lumbar spine (L2-4) BMD in these patients [36,37]. These findings suggest that smoking may contribute to bone loss by modulating monocyte activity. Our mediation analysis also demonstrated that MONs can mediate the relationship between serum cotinine concentrations and total BMD.

Furthermore, harmful substances in tobacco (such as nicotine and tar) may directly or indirectly affect the activity of LYMs, leading to immune dysfunction [38]. When LYM function is disrupted or its quantity is abnormal, it can cause a chronic inflammatory state in the body, releasing a large amount of inflammatory mediators, which in turn affect changes in bone mass and bone density. However, the mechanism is not yet fully understood. LYM can promote the expression of transcripts for cytokines produced by osteoclasts, like RANKL, IL-6, TNF-α, IL-1β, and IFN-γ, thereby leading to a decrease in bone density [39]. Additionally, research has confirmed that B LYM lineage cells can express osteoclast differentiation factor/RANKL to facilitate osteoclast differentiation, thereby triggering OP [40]. Notably, Peng *et al.* find that the OP group exhibits notably lower absolute counts of total T LYMs and CD8 + T LYMs relative to the non-OP group. As the count of CD8+ T LYMs decreases, the BMD of the right femoral neck also evidently decreases [41]. Similarly, Monaco *et al.* also find that the total LYM count is notably positively tied to femoral BMD in healthy postmenopausal women [42]. In contrast, this study discovers that LYM does not act as a mediator between cotinine and total BMD. Smoking not only increases PLT count but also leads to PLT dysfunction [43]. PLTs originate from the cytoplasmic fragmentation of megakaryocytes in the bone marrow, and their influence on BMD is complex, encompassing both positive and negative effects. PLTs assist in bone formation, where PLT-derived growth factors enhance bone formation by affecting cell proliferation, chemotaxis, differentiation, and extracellular matrix synthesis [44]. It is also found that the mean PLT volume and PLT distribution width levels in the OP group are lower than those in the normal BMD group, indicating that PLT function is linked to bone mineralization [45]. Conversely, Zhang *et al.* demonstrate that an increase in PLT count is tied to a decrease in BMD in the adult population of the U.S. [46]. Moreover, PLTs are capable of expressing RANKL, which binds to RANK on the surface of osteoclast precursor cells, stimulating osteoclast formation and activation, thus increasing bone resorption. Osteoprotegerin (OPG) is a natural inhibitor of RANKL that can competitively bind to RANKL, preventing its activation of osteoclasts. PLTs influence the dynamic balance of bone resorption and formation by modulating the balance between RANKL and OPG [47]. In conclusion, smoking-induced systemic stress and inflammation response result in elevated levels of these inflammatory markers (LYMs, NEUs, MONs, and PLTs), while they can affect bone density through complex mechanisms.

Recent studies have found that a new composite inflammation index can be calculated using blood inflammatory markers, including LYMs, NEUs, MONs, and PLTs. For example, the MON-to-LYM ratio (MLR), NEU-to-LYM ratio (NLR), and PLT-to-LYM ratio (PLR) are likely closely tied to systemic inflammation and immune response [48]. The measurement of these biomarkers can be a strong predictor of OP. According to Zhang *et al.*, high levels of PLR, NLR, and MLR are tied to a higher OP risk. Particularly, NLR is a strong indicator of OP risk, thus representing a valuable and convenient inflammatory marker for predicting OP risk [49]. One study has discovered that NLR is positively linked to lumbar bone density, while PLR is negatively tied to lumbar BMD, implying that PLR might be a potential inflammatory predictor for OP [50]. According to Jiang *et al.*, NLR exhibits a negative relationship with procollagen type 1 N-terminal propeptide (P1NP) and β-C-terminal telopeptide of type 1 collagen (β-CTX), while PLR also exhibits a negative relationship with P1NP and β-CTX [51].

Several limitations should be acknowledged in this study. First, given the cross-sectional design of this study, it can only identify associations between serum cotinine concentrations, inflammatory markers, and total BMD, but cannot establish causality. Future longitudinal studies are needed to further explore the causal mechanisms among these variables.

Second, LYMs, MONs, NEUs, and PLTs were selected as inflammation-related variables mainly due to their important roles in bone metabolism and their ability to comprehensively reflect inflammatory status. NEUs and MONs are the main sources of pro-inflammatory cytokines in the bone microenvironment, which can directly activate osteoclasts. PLTs contribute to the regulation of bone remodelling by releasing growth factors such as TGF-β and PDGF, and changes in PLT counts are closely related to the progression of OP.

Third, data on LYMs, MONs, NEUs, and PLTs in NHANES were available across more survey cycles than classical markers such as CRP, IL-6, and TNF-α, with IL-6 and TNF-α measured only in limited cycles. This broad data availability allowed for a sample size of more than 20,000 participants, significantly enhancing the statistical power of this study. While we fully acknowledge the importance of classical inflammatory markers (such as CRP, IL-6, and TNF-α), the selection of inflammatory variables in this analysis was primarily driven by these practical considerations.

Given these limitations, future studies should consider incorporating classic inflammatory markers such as CRP, IL-6, and TNF-α to further validate and complement the findings of this study.

## Conclusion

Smoking contributes to the reduction of BMD in the human body. Its influence on BMD is partly mediated by inflammatory markers such as MONs and NEUs. PTLs can somewhat reduce the effect of cotinine on total BMD.

## Supporting information

S1 File Strobe checklist. (DOCX)
